# Blood Pressure over Height Ratios: Simple and Accurate Method of Detecting Elevated Blood Pressure in Children

**DOI:** 10.1155/2012/253497

**Published:** 2012-04-08

**Authors:** Ovidiu Galescu, Minu George, Sudhakar Basetty, Iuliana Predescu, Anil Mongia, Svetlana Ten, Amrit Bhangoo

**Affiliations:** ^1^Division of Pediatric Endocrinology Infants and Children's Hospital of Brooklyn at Maimonides & Children and Hospital at Downstate, State University of New York, Brooklyn, NY 11219, USA; ^2^Division of Pediatric Nephrology, Children's Hospital at Downstate, State University of New York, Brooklyn, NY 11219, USA

## Abstract

*Background*. Blood pressure (BP) percentiles in childhood are assessed according to age, gender, and height. *Objective*. To create a simple BP/height ratio for both systolic BP (SBP) and diastolic BP (DBP). To study the relationship between BP/height ratios and corresponding BP percentiles in children. *Methods*. We analyzed data on height and BP from 2006-2007 NHANES data. BP percentiles were calculated for 3775 children. Receiver-operating characteristic (ROC) curve analyses were performed to calculate sensitivity and specificity of BP/height ratios as diagnostic tests for elevated BP (>90%). Correlation analysis was performed between BP percentiles and BP/height ratios. *Results*. The average age was 12.54 ± 2.67 years. SBP/height and DBP/height ratios strongly correlated with SBP & DBP percentiles in both boys (*P* < 0.001, *R* ^2^ = 0.85, *R* ^2^ = 0.86) and girls (*P* < 0.001, *R* ^2^ = 0.85, *R* ^2^ = 0.90). The cutoffs of SBP/height and DBP/height ratios in boys were ≥0.75 and ≥0.46, respectively; in girls the ratios were ≥0.75 and ≥0.48, respectively with sensitivity and specificity in range of 83–100%. *Conclusion*. BP/height ratios are simple with high sensitivity and specificity to detect elevated BP in children. These ratios can be easily used in routine medical care of children.

## 1. Introduction

 Hypertension in children and adolescents is defined as systolic BP (SBP) and/or diastolic BP (DBP) above the 95th percentile. BP between the 90th and 95th percentile is designated “high normal” or “prehypertensive” [1]. Currently the available data to quantify hypertension in children is in the form of a table reference matched by sex, age, and height percentile that identifies the BP as 50th, 90th, 95th, and 99th percentiles [2]. 

In recent years the prevalence of hypertension in the pediatric population in the United States has been increasing [3]. Children and adolescents with primary hypertension are frequently overweight or obese. The prevalence of hypertension increases progressively with increasing body mass index (BMI), and hypertension is detectable in over 30% of overweight children with BMI above 95th percentile [4]. The increase in the prevalence of childhood obesity and its strong association with hypertension make both prehypertension and hypertension a major health problem in the young [5].

Hypertension in childhood and adolescence can lead to hypertension as adults [6, 7]. Although hypertension in childhood has been thought of as an independent risk factor for CVD (cardiovascular vascular disease), there are several studies demonstrating the association of hypertension with other risk factors such as hypercholesterolemia, hypertriglyceridemia, low HDL cholesterol, truncal (central) obesity, hyperinsulinemia, and metabolic syndrome [8, 9].

The criteria for diagnosis of hypertension in children and adolescents can be complex and challenging to apply in clinical practice because of age-, gender-, and height-specific reference values [10]. Both hypertension and prehypertension though prevalent and increasing in incidence remain widely undiagnosed [11]. In 2010 Lu et al. evaluated, for the first time, the feasibility and accuracy of the systolic blood pressure-to-height ratio (SBP/height ratio) and the diastolic blood pressure-to-height ratio (DBP/height ratio) and proposed the optimal thresholds of SBP/height ratio and DBP/height ratio for identifying hypertension in Chinese adolescents. They concluded that the BP/height ratios are simple, inexpensive, and accurate indices for identification of hypertension in adolescents [12]. Subsequently BP/height ratios and cutoffs were also proposed for Italian and Nigerian adolescents [13, 14].

In order to validate the BP/height ratios in US children, similar studies needed to be repeated and studied further. Therefore, this formed the basis of hypothesis of our study. In this study we tested that simple BP/height ratios can be used as an accurate clinical tool to identify children and adolescents at risk for hypertension. For this purpose we have reported the simplified ratios of BP/height as an accurate detection method of elevated BP. We also report the correlation of BP/height ratios with commonly used BP percentiles.

## 2. Materials and Methods

### 2.1. Data Collection

We analyzed data on 3775 children from NHANES 2007-2008 (*National Health and Nutrition Examination Survey)*. Data on age, gender, ethnicity, height, weight, and waist circumference was analyzed [13]. Systolic blood pressure (SBP) and diastolic blood pressure (DBP), were reported to be measured by a standard protocol used by NHANES [14]. Body mass index (BMI) was calculated using the standard formula. BP percentiles were calculated according to the standard formula provided in the following referenced manuscript [2].

### 2.2. Statistical Analysis

Receiver-operating characteristic (ROC) curve analyses were performed to calculate sensitivity and specificity of SBP/height and DBP/height ratios as diagnostic tests for elevated, that is, >90th percentile of SBP and DBP, respectively. Correlation analysis was performed between SBP percentile and SBP/height ratio. Similar correlation was studied for DBP percentiles and DBP/height ratio. ROC analyses and curve generation were performed using Analyse-it Method Evaluation edition. For data analysis and interpretation we used SPSS v.19.

## 3. Results

 Data was generated from 1982 boys aged 12.5 ± 2.6 years old and 1783 girls aged 12.5 ± 2.6 years old. The distribution by ethnicity as defined in the NHANES Demographics Variable List was accessed. The various ethnicities were as follows: 1069: Mexican Americans, 383: other Hispanics, 1083: non-Hispanic Whites, 1047: non-Hispanic Blacks, and 183-other Races. The BMI percentile in girls from all ethnic groups was  67 ± 29  (Mean ± SD). Approximately 23 percent of girls had BMI greater than 95th percentile. The BMI percentile in boys from all ethnic groups was  66 ± 30  (Mean ± SD). The proportion of boys with BMI greater than 95th percentile was approximately 24 percent. The proportion of overweight girls and boys with BP reading above 90th percentile was 11% and 10% respectively. The proportion of BP above 90th percentile in all girls and boys was 6.3% and 5.2%, respectively.

**Figure 1:**
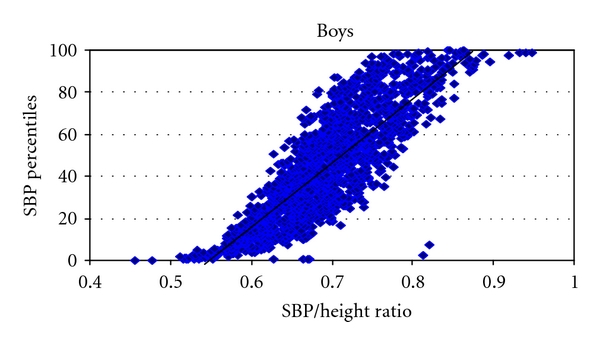
The graph depicts the strong correlation of systolic blood pressure percentiles (SBP Percentiles), on the *Y* Axis and the systolic blood pressure over height ratio (SBP/height ratio) on the *X* Axis in boys *P* < 0.001, *R* ^2^ = 0.85, SBP: systolic blood pressure.

**Figure 2:**
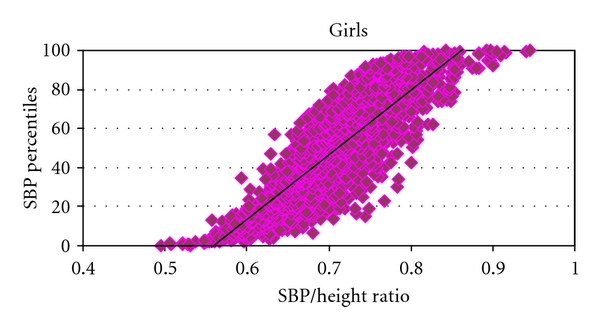
The graph depicts the strong correlation of systolic blood pressure percentiles (SBP Percentiles), on the *Y* Axis and the systolic blood pressure over Height ratio (SBP/height ratio) on the *X* Axis in girls *P* < 0.001, *R* ^2^ = 0.80. SBP: Systolic Blood Pressure.

SBP/height ratio strongly correlated with SBP percentiles in both boys (Figure 1; *P* < 0.001, *R* ^2^ = 0.85) and girls (Figure 2; *P* < 0.001, *R* ^2^ = 0.85). Similar results were obtained for DBP/height ratio and DBP percentiles: boys' *P* < 0.001, *R* ^2^ = 0.86 and girls' *P* < 0.001, *R* ^2^ = 0.90. ROC analysis showed a very steep progression of sensitivity and specificity above certain cutoff values. The cutoffs of elevated SBP/height and DBP/height ratios in boys were ≥0.75 and ≥0.46; in girls the ratios were ≥0.75 and ≥0.48, respectively. Above the cutoff values of the ratios the sensitivity and specificity of detection of elevated BP (greater than 90th percentile) was very high as shown in (Table 1). There was no significant difference of BP/height ratios in various ethnicities as demonstrated by an inter-group weighted *t* test (data not shown).

The accuracy of this determination of ratios above the 95th percentile is diminished by the low number of children. In this cohort there were only 50 boys (2.6%) who had SBP percentiles above 95th percentile and only 6 (0.3%) had DBP above this range. Similarly 46 girls (2.6%) had SBP percentiles above 95th percentile and only 7 (0.4%) had DBP percentiles in the hypertensive range.

## 4. Discussion

Hypertension in the pediatric and adolescent population may remain undiagnosed. Although the BP charts for age, gender and height are readily available, they are generally not used in a busy pediatric practice. The diagnosis of hypertension may be missed in some children [10]. In 2008 Cunningham reviewed data on 14,187 children and adolescents. Overall, 507 children fulfilled the criteria for hypertension, of whom 74% were undiagnosed. Prehypertension was documented in 485 individuals, but 89% had not been previously diagnosed. [11] The authors concluded that the current practice of identifying the BP percentiles from lengthy tables can be cumbersome, ineffective, and can often miss the diagnosis of hypertension in children, which can become detrimental to their long-term health [11].

In this study we have shown that BP/height ratios are simple and accurate method of diagnosing elevated blood pressure. Our findings are in complete agreement with the preceding studies on this matter [12–14]. The authors in that study clearly demonstrated the feasibility and accuracy of the SBP/height and DBP/height ratios in their cohort of Chinese, Italian, and Nigerian adolescents [12–14]. In our study the cutoff for SBP/height ratio for both boys and girls is 0.75. The cutoffs for DBP/height ratio in boys and girls are 0.46 and 0.48, respectively (Table 1). The sensitivities and specificities of these ratios are from 83% to 100% in identifying elevated BP above the 90th percentile. Our study is the first to report the BP/height ratio for prehypertension, which can be used as a reliable tool not only for diagnosis but also screening for high BP in ambulatory care setting in children. Also, there were no significant differences of BP/height ratios in various ethnicities as demonstrated by intergroup weighted *t* tests. Therefore, we have proven for the first time that these ratios can be used universally in most ethnic groups represented in USA In addition, we have shown that SBP/height and DBP/height ratios strongly correlated with their respective SBP and DBP percentiles in both boys and girls (Figures 1 and 2).

Our study has a large cohort of subjects derived from the NHANES database. Hence the ROC analysis for the calculated cutoffs generates good power and validation (Table 1). In this cohort the incidence of BP above 95th percentile was low (below 3% in both boys and girls). Therefore it is our belief that more studies need to be done with looking at a larger cohort of hypertensive children to assess the reliability and reproducibility of the BP/Height ratios. Although NHANES data focuses on US children and adolescents without any particular pathology, the large numbers of subjects in this study make it possible to extrapolate the results at a population level. Also this study is based on cross-sectional data and does not imply target organ damage, which would need standard diagnostic techniques such as echocardiogram.

## 5. Conclusions

BP/height ratio is a simple, excellent screening and diagnostic tool with high sensitivity and specificity to detect elevated BP. The BP/height ratio can be easily used in routine medical care of children. The BP/height ratios also correlated with the corresponding BP percentiles in both genders. More studies are needed in patients with proven hypertension to validate the use of this proposed BP/height ratio in conditions with severe elevations of blood pressure.

## Figures and Tables

**Table 1:** ROC analysis of SBP and DBP in boys and girls. Area under the curve (AUC) and corresponding *P* values are depicted with the associated confidence intervals for the calculated cutoff values of the BP/height ratios greater than 90th percentile. Correlation of the BP/height ratios with their respective BP for each cutoff value is quantified by sensitivity and specificity percentages.

	Cutoff BP/height ratios	AUC	*P* value	CI	*N*	Sensitivity (%)	Specificity (%)
Boys							
SBP > 90%	≥ 0.75	0.95	<0.0001	0.94–0.96	1979	95	83
DBP > 90%	≥ 0.46	0.95	<0.0001	0.87–1	1972	92	91.5

Girls							
SBP > 90%	≥ 0.75	0.96	<0.0001	0.94–0.97	1765	97.2	83.3
DBP > 90%	≥ 0.48	0.99	<0.0001	0.98-0.99	1762	100	95.5

SBP: systolic blood pressure, DBP: diastolic blood pressure, AUC: area under the curve, CI: confidence interval, and *N*: number of children in category.

## Abbreviations

CDC: Centers for Disease Control and Prevention
NHANES: National Health and Nutrition Examination Survey
ROC: Receiver-operating characteristic
CVD: Cardiovascular vascular disease.
